# Ethanol-Gas-Sensing Performances of Built-in ZrO_2_/Co_3_O_4_ Hybrid Nanostructures

**DOI:** 10.3390/s23239578

**Published:** 2023-12-02

**Authors:** Madiha Khan, Angelo Ferlazzo, Mozaffar Hussain, Enza Fazio, Carmelo Corsaro, Angela Maria Mezzasalma, Giovanni Neri

**Affiliations:** 1 Department of Engineering, University of Messina, C.da Di Dio, 98166 Messina, Italy; madihakhan@students.au.edu.pk; 2 Department of Physics, Air University, PAF Complex, E-9, Islamabad 4400, Pakistan; 3 Department of Chemical Sciences, University of Catania, Viale A. Doria 6, 95125 Catania, Italy; angelo.ferlazzo@unict.it; 4 Department of Mathematical and Computational Sciences, Physical Sciences and Earth Sciences, University of Messina, Viale F. Stagno D’Alcontres 31, 98166 Messina, Italy; enza.fazio@unime.it (E.F.); ccorsaro@unime.it (C.C.); angelamaria.mezzasalma@unime.it (A.M.M.)

**Keywords:** gas sensors, ethanol, zirconium oxide, cobalt oxide, heterostructure, nanocomposite

## Abstract

The development of novel nanomaterials as highly efficient gas-sensing materials is envisaged as one of the most important routes in the field of gas-sensing research. However, developing stable, selective, and efficient materials for these purposes is a highly challenging task requiring numerous design attempts. In this work, a ZrO_2_/Co_3_O_4_ composite is reported, for the first time, as a gas-sensing material for the detection of ethanol. The sensitive and selective detection of ethanol gas at 200 °C has been demonstrated for the ZrO_2_/Co_3_O_4_ (0.20 wt%/0.20 wt%)-based sensor. Furthermore, the sensor showed a very low response/recovery time of 56 s and 363 s, respectively, in response to a pulse of 20 ppm of ethanol and good stability. The interesting gas-sensing property of ZrO_2_/Co_3_O_4_ can be ascribed to both the porous structure, which facilitates the interaction between the target gas and the sensing site, and the p–p-junction-induced built-in electric field. These results indicate that the ZrO_2_/Co_3_O_4_ composite can serve as a heterostructured nanomaterial for the detection of ethanol gas.

## 1. Introduction

Conductometric gas sensors based on a metal oxide (MOX) sensing layer are simple devices that are able to detect dangerous and toxic gases [1]. Their performance depends on the structure of the carefully designed materials used as the sensing layer [2,3]. Heterostructured metal oxides such as Co_3_O_4_-decorated Fe_2_O_3_ [4], Co_3_O_4_/SnO_2_ [5], Y_2_O_3_-doped ZrO_2_, etc., [6] have been reported to display better performances in comparison to pure metal oxides (Co_3_O_4_, CuO, NiO, etc.) [7], such as a high response, high conductivity, and low working temperature. Therefore, designing a heterostructured material is advantageous for improving the gas-sensing performance. For efficient detection, the designed metal oxide nanomaterials should also have well-structured active sites, a large surface area, porosity, and a chemically stable structure [8,9].

Recently, Fe_2_O_3_ nanocomposites (FeNCs) [4], NiO-loading Co_3_O_4_ NCs [10], and Co_3_O_4_-CuO heterostructures [11] have been used for gas-sensing applications. The combinations of a high surface area, excellent electrical conductivity, and porosity, which facilitates the diffusion of gases, increase the performances of the modified metal oxides [12]. In this paper, we investigate, for the first time, the sensing properties of Co_3_O_4_-ZrO_2_ heterostructures. ZrO_2_ nanoparticles are widely used in catalysis, energy, and environment fields, due to their thermal stability, high specific surface area, and optical and electrical properties [13,14].

However, recently, ZrO_2_ nanoparticles have drawn the attention of researchers due to their improved gas-sensing performance [15], especially for designing electrochemical gas sensors. The use of ZrO_2_ for electrochemical gas sensors (e.g., as potentiometric YSZ oxygen sensors) is well-known, while very few papers report the use of pure or doped ZrO_2_ in conductometric gas sensors. Both of these solid-state sensors offer high sensitivity, are robust, and are suitable for the detection of a wide variety of gases. Moreover, conductometric sensors have a high tolerance to extreme ambient conditions and corrosive environments, making them a choice for monitoring gases in hot, dry climates.

For conductometric gas sensors, pure ZrO_2_ has some limitations because of its insulating nature and, therefore, presents a very high resistance, which hinders the measurements in the usual range of sensor’s operating temperature. Suitable microstructural modifications, by doping or heterojunction formations, are necessary to improve the electrical characteristics of zirconia for gas-sensing applications [6].

Yan and coworkers reported the detection of NO_2_ at room temperature using surface-microstructure-controlled ZrO_2_ nanoparticles [16]. John and coworkers synthesized a metal/metal oxide mixture within a yttria-stabilized zirconia oxygen-conducting ceramic superstructure and found that the material shows interface oxygen at the target application temperature [17]. Zeng and coworkers developed a compact yttria-stabilized zirconia NOx sensor with a dual-functional Co_3_O_4_/NiO-sensing electrode [18]. Hence, ZrO_2_ nanoparticles can be considered as a highly efficient gas-sensing material for a variety of gases. On the other hand, zirconium oxide, as a support material [19], offers striking features, due to its hydrophilic nature.

Based on the above facts, here, we report results based on the exploration of a new Co_3_O_4_-ZrO_2_ heterostructure, demonstrating its promising ethanol-sensing ability. The detection of ethanol at low temperatures is top-priority research amongst many scientists and engineers because this dangerous gas can cause depression, leading to sedation, slurred speech, impaired judgment, uninhibited behavior, and euphoria. The detection of ethanol has drawn extensive attention due to ingestion, inhalation, or by skin absorption [20], and hence a suitable sensor is essentially required. A number of metal oxides, noble metals, single- and multi-wall carbon nanotubes, and graphene oxide have been exploited for the detection of ethanol [21,22]. The composites of transition metal oxides provide better gas-sensing activity, good selectivity, and a fast response. Therefore, in most cases, materials based upon metal oxides serve as the main transport component for electrode fabrication [23].

In the present work, the sol-gel method is used for the synthesis of cobalt-oxide-decorated zirconium oxide (ZrO_2_-Co_3_O_4_) nanocomposites and we have exploited the as-prepared heterostructure for the sensitive and selective detection of ethanol.

## 2. Materials and Methods

### 2.1. Reagents and Chemicals

The cobalt oxide nanoparticles (Co_3_O_4_) were prepared by using nitrates of pure grade (99.99%) and zirconium dioxide nanoparticles (ZrO_2_ NPs) (99.99%) acquired from Sigma Aldrich. The ZrO_2_/Co_3_O_4_ (0.2 wt%: x = (0.0, and 0.2 wt%)) nanocomposites (NC) were synthesized. Ammonia, hydrogen, acetone, nitrogen, ethanol, and hydrogen were purchased from ASL (Messina, Italy). The codes given to the samples were as follows: C for pure cobalt oxide NPs, Z for pure zirconium dioxide, and CZ for ZrO_2_/Co_3_O_4_ (0.20/0.20 wt%) NCs.

### 2.2. Synthesis of ZrO_2_/Co_3_O_4_ Junctions

**Figure 1:**
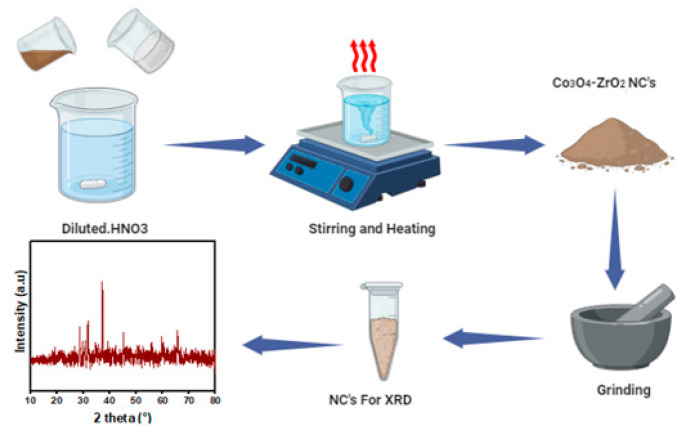
Sol-gel preparation of the ZrO_2_/Co_3_O_4_ nanocomposite and its characterization step via XRD analysis of the obtained powders.

The ZrO_2_/Co_3_O_4_ nanocomposites were prepared using a two-step procedure. First, pure Co_3_O_4_ nanoparticles (NPs) were prepared using the precipitation method. To prepare the ZrO_2_/Co_3_O_4_ (0.20/0.20 wt%) nanocomposites (NCs), 0.481 g (0.20 wt%) of Co_3_O_4_ NPs powder was dissolved in 5 mL of diluted HNO_3_. To achieve homogeneity, the solution was stirred at room temperature for 30 min using a hotplate. The same procedure was adopted to prepare the solution of ZrO_2_ NPs, using 0.246 g (0.20 wt.%) of ZrO_2_ NPs dissolved in diluted HNO_3_ and stirred for 30 min. When the single homogeneous solutions were ready, we mixed the pristine solutions. A scheme of the synthesis procedure is shown in Figure 1 [24].

Subsequently, the beakers containing the as-obtained solution were placed on a hotplate at 300 °C until a gel was obtained. The product was removed from the hotplate after the substance had completely burned. Then, the nanocomposite powder was ground using a mortar and pestle. The result was a blackish powder.

### 2.3. Sample Characterization

The synthesized materials were analyzed using the following techniques: powder X-ray diffraction (XRD), Raman and FT-IR spectroscopies, and scanning electron microscopy (SEM) equipped with an energy-dispersive X-ray (EDX) spectrometer. To analyze the surface morphologies and the samples’ elemental composition, we used a Zeiss microscope operating at the acceleration voltage of 20 kV. The XRD patterns were recorded with a D2 phaser Bruker X-ray diffractometer using the Cu Kα line (0.159 nm) in the 10–80° 2θ range. Raman spectra were collected using the XploRA micro-Raman setup (Horiba, Kyoto, Japan) equipped with a diode laser emitting at 532 nm. The scattered signal was collected with a 50× focal length objective, dispersed by an 1800 mm/line grating, and collected with a Peltier CCD detector. The measurements were performed at room temperature. The Fourier transform infrared spectroscopy (FT-IR) spectra were acquired with a Perkin Elmer spectrometer equipped with a universal ATR sampling accessory.

### 2.4. Sensor Fabrication and Gas-Sensing Measurements

The solid-state gas sensor device we used was composed of a thick layer of the sensing material deposited on a patterned ceramic substrate [6]. The procedure for the sensor fabrication was as follows: First, the powder material was dispersed in deionized water, and then the paste obtained was put on the ceramic substrate made in alumina (3 mm × 6 mm), supplied with interdigitated Pt electrodes and heating elements. Finally, the deposited layer was dried using a hotplate to favor water evaporation, and, to stabilize the microstructure of the sensing film, a thermal treatment at 400 °C for 2 h was carried out.

A homemade gas-sensing setup was used for the gas-sensing measurements. The measurements were performed under a dry air total stream of 200 sccm, collecting the sensors’ resistance data in the four-point mode. The concentration of target gas (ethanol) varied from 10 to 100 ppm. A multimeter data acquisition unit, Agilent 34970A, was used for this purpose, while a dual-channel power supplier instrument, Agilent E3632A (Agilent, Santa Clara, CA, USA) gave the input to the sensors and performed the measurements at super-ambient temperatures.

The sensor response, R, was determined as the ratio of Ra (resistance of the gas sensor in dry synthetic air) to Rg (resistance of the injected gas sensor), R = Ra/Rg [4].

## 3. Results

### 3.1. Structure and Morphology Studies

**Figure 2:**
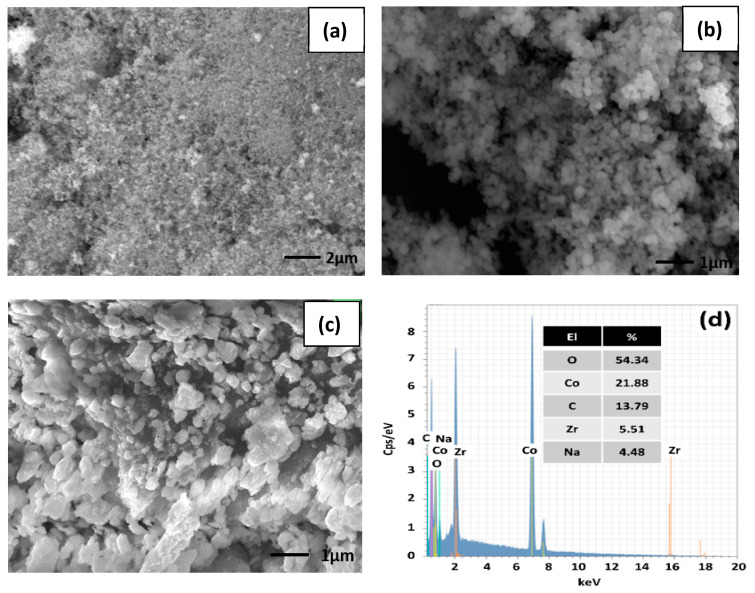
(**a**–**c**) SEM images for pure Co_3_O_4_, pure ZrO_2_, and the ZrO_2_/Co_3_O_4_ composite; (**d**) EDX spectrum of the ZrO_2_/Co_3_O_4_ sample and in the inset of the percentage of the atomic species.

Figure 2a–c shows the SEM images for all of the investigated samples. The samples’ surfaces are characterized by a highly porous structure. This porosity nature facilitated gas diffusion, thereby enhancing the sensing performances.

In fact, as reported in the next section, the gas-sensing performance benefits from the samples’ high porosity, large specific surface area, and the remarkable capabilities of surface-adsorbed oxygen. The morphology changes observed in the ZrO_2_/Co_3_O_4_ nanocomposite, i.e., an increase in the average sample grain size with respect to the pristine ZrO_2_ and Co_3_O_4_ materials, is likely due to the different synthesis procedures (see Section 2.2.) adopted for the pristine metal oxides and the composite Co_3_O_4_/Fe_2_O_3_ heterostructure.

On the other hand, the EDX analysis (Figure 2d) confirmed the presence of the atomic Co, O, and Zr species in the structure of the nanocomposite, with the expected ratio, apart from the presence of C and Na, due to preparation residues or ambient contamination.

**Figure 3:**
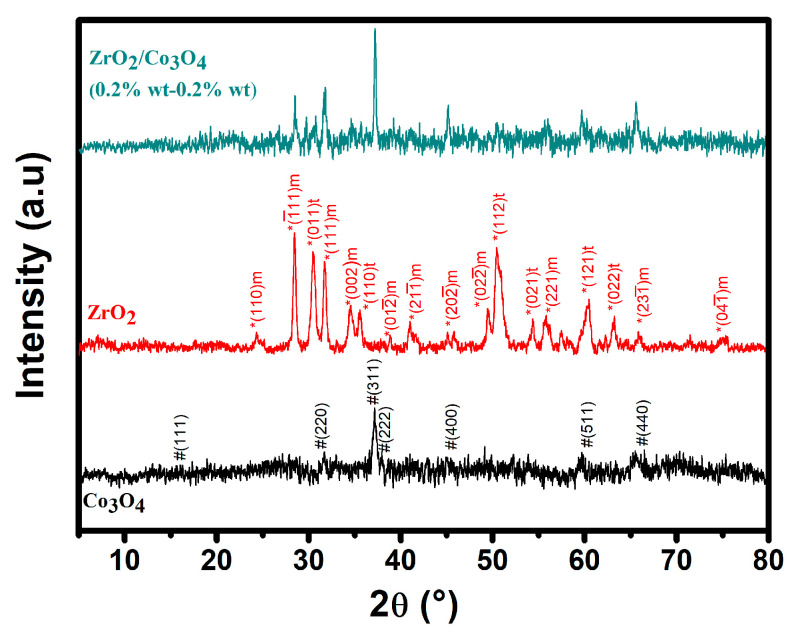
XRD patterns of Co_3_O_4_, ZrO_2_, and the ZrO_2_/Co_3_O_4_ composite. Symbols “*” and “^#^” are related to the reflections attributed to the pure ZrO_2_ and Co_3_O_4_ phase, respectively.

As shown in Figure 3, the XRD pattern of the pure samples and of the final ZrO_2_/Co_3_O_4_ composite exhibited the well-developed Bragg diffraction peaks of a typical cubic system (JCPDS No. 74–2120). Specifically, we were able to assign all of the characteristic diffraction peaks of the ZrO_2_ and Co_3_O_4_ nanoparticles. The diffraction pattern of the final ZrO_2_/Co_3_O_4_ composite, indeed, contains some characteristic features of both of the systems. In particular, the peaks in the range of 28° < 2θ < 38° can be ascribed to the (220) and (311) reflections of Co_3_O_4_ [25] and to the (-111) m, (011) t, and (111) m reflections of the monoclinic (m) and triclinic (t) phases of ZrO_2_ (see Figure 3 and JCPDS No. 37-1484 27 [26]). In addition, the application of the Scherrer’s equation to the (311) plane diffraction peak yields a crystal size of ~40 nm. The diffraction peaks of the ZrO_2_/Co_3_O_4_ composite, occurring at about 2θ = 45°, 60°, and 65°, correspond to the superposition of the reflections from both of the pure samples.

The Raman spectrum of the ZrO_2_ powder shows vibration bands at about 334, 344, 381, 480, 557, 617, and 632 cm ^−1^, which are assigned to the monoclinic phase. Meanwhile, the vibration bands at 315, 462, and 642 cm^−1^ belong to tetragonal phase of ZrO_2_ [27]. The Raman spectrum of the pure Co_3_O_4_ powder shows the modes predicted by the group theory for Co_3_O_4_, consisting of Eg + 2F1g + F2g + A1g. The Raman mode at 684.5 cm^−1^ (A1g) is attributed to the characteristics of the octahedral sites, and the Eg and F2g modes are likely related to the combined vibrations of the tetrahedral site and the octahedral oxygen motions. Furthermore, the Raman bands at 470, 510, 608, and 670 cm^−1^ are assigned to the Co_3_O_4_ spinel structure, confirming the XRD data [28]. Some of these features can be observed in the Raman spectrum of the ZrO_2_/Co_3_O_4_ nanocomposite. In addition, the new bands centered at about 520 and 690 cm^−1^ are likely due to the lattice distortion of the spinel structure induced by the presence of Zr in the Co_3_O_4_ matrix (by weaking the Co–O bonds), similarly to those observed in Zr-doped Co_3_O_4_ catalysts [29].

**Figure 4:**
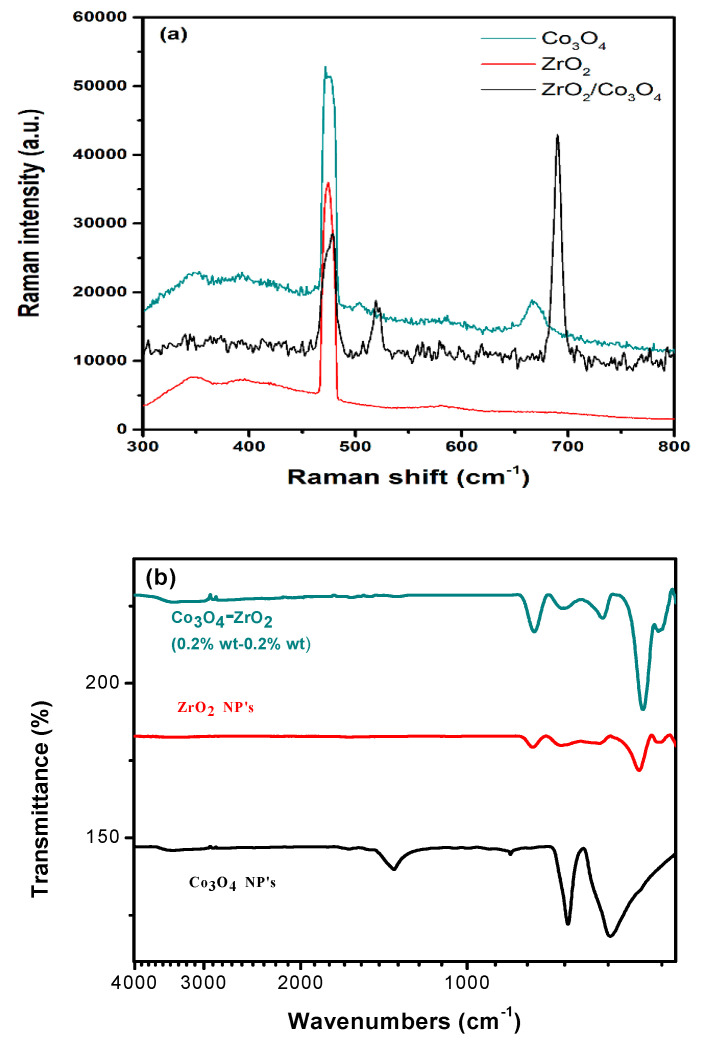
(**a**) Raman and (**b**) FT-IR spectra of pure ZrO_2_ (red line), pure Co_3_O_4_ (black line), and the ZrO_2_/Co_3_O_4_ nanocomposite (blue line).

The FT-IR spectra of the Co_3_O_4_, ZrO_2_, and ZrO_2_/Co_3_O_4_ nanocomposite samples, collected in the 4000–400 cm^−1^ range, are shown in Figure 4b. The Co_3_O_4_ FT-IR spectrum shows a peak at 560 cm^−1^, corresponding to the Co^3+^-O stretching in the octahedral hole, and a peak at 660 cm^−1^, ascribed to the stretching vibrations in the Co^2+^-O in the tetrahedral hole, typical of the single-phase face-centered cubic Co_3_O_4_ structure [30,31]. The band centered at about 1360 cm^−1^ is ascribed to C-O stretching, while the weak peak centered at about 3400 cm^−1^ belongs to the OH-stretching modes of the adsorbed water molecules [32]. The spectral region at the highest wavenumbers is multiplied by 20 for clarity. The ZrO_2_ FT-IR spectrum exhibits the main characteristic bands centered at about 750 cm^−1^, 580 cm^−1^, and 490 cm^−1^, corresponding to the stretching vibration of Zr–O of ZrO_2_ [33,34,35]. As in the Raman case, some of the FT-IR features of the pure compounds characterize the spectrum of the ZrO_2_/Co_3_O_4_ sample.

### 3.2. Electrical and Gas-Sensing Measurements

The sensing properties of the pure Co_3_O_4_, pure ZrO_2_, and the ZrO_2_-Co_3_O_4_ nanocomposite sensors towards ethanol were investigated here, for the first time, to our knowledge. In order to find the best operating conditions, the sensors were preliminary exposed to 20 ppm of ethanol at different operating temperatures. The operating temperature was monitored and controlled with a heater located at the rear of the ceramic platform that was used as the sensing substrate. The range of temperature investigated was between 50 °C and 300 °C; however, at the lower temperatures, restrictions were imposed by the very high resistance registered. In particular, the ZrO_2_-based sensor displayed very high resistance values in dry air (e.g., 1.9 × 10^11^ ohm at 200 °C and 6.9 × 10^8^ ohm at 400 °C). The same findings were displayed by the Co_3_O_4_ sensor.

**Figure 5:**
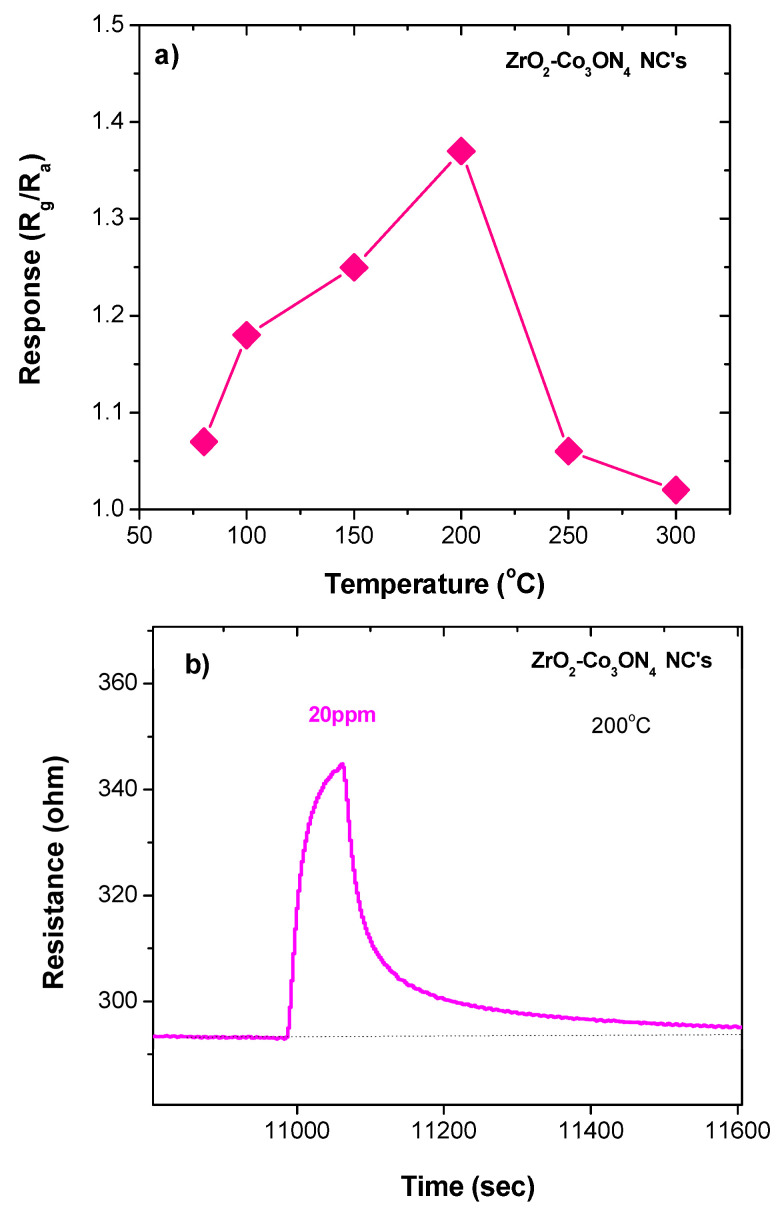
(**a**) Response at different temperatures of the composite sensor; (**b**) transient response curve at 200 °C.

Figure 5a shows the resistance change (response) for the ZrO_2_/Co_3_O_4_-nanocomposite-based sensor at temperatures of 50 °C, 80 °C, 100 °C, 150 °C, 200 °C, 250 °C, and 300 °C. It is quite common for the response of semiconductor sensors to improve with the increase in the operating temperature, followed by a decrease in response, with a further increase in temperature, as was observed for the present ZrO_2_/Co_3_O_4_ nanocomposite sensor. This behavior can be attributed to the different thermal activation of the adsorption/reaction/desorption processes, which, together with the charge transfer at the electrode-sensing layer, are essential pathways for the response of the conductometric sensors.

At the optimal temperature of 200 °C, the response of the sensor gradually increased after ethanol injection and then quickly fell back to the initial value, due to the rise in temperature (see Figure 5b). This indicates the fast and reversible response and recovery behavior of the sensor. Furthermore, based on the response observed here to ethanol, a reducing gas, which occurs through an increase in the baseline resistance, a p-type behavior was envisaged. Zirconia has been reported as a semiconductor with a wide band gap energy of ~5.0 eV, both with n-type [36] and p-type behavior [37]. Considering that the n- or p-type semiconducting properties of metal oxides are largely dependent on many variables, such as temperature, grain size, the nature of the gas surrounding the sensing material, etc., a deeper analysis is necessary to formulate a more precise hypothesis on the semiconducting characteristics of our ZrO_2_ and ZrO_2_-based nanocomposite.

**Figure 6:**
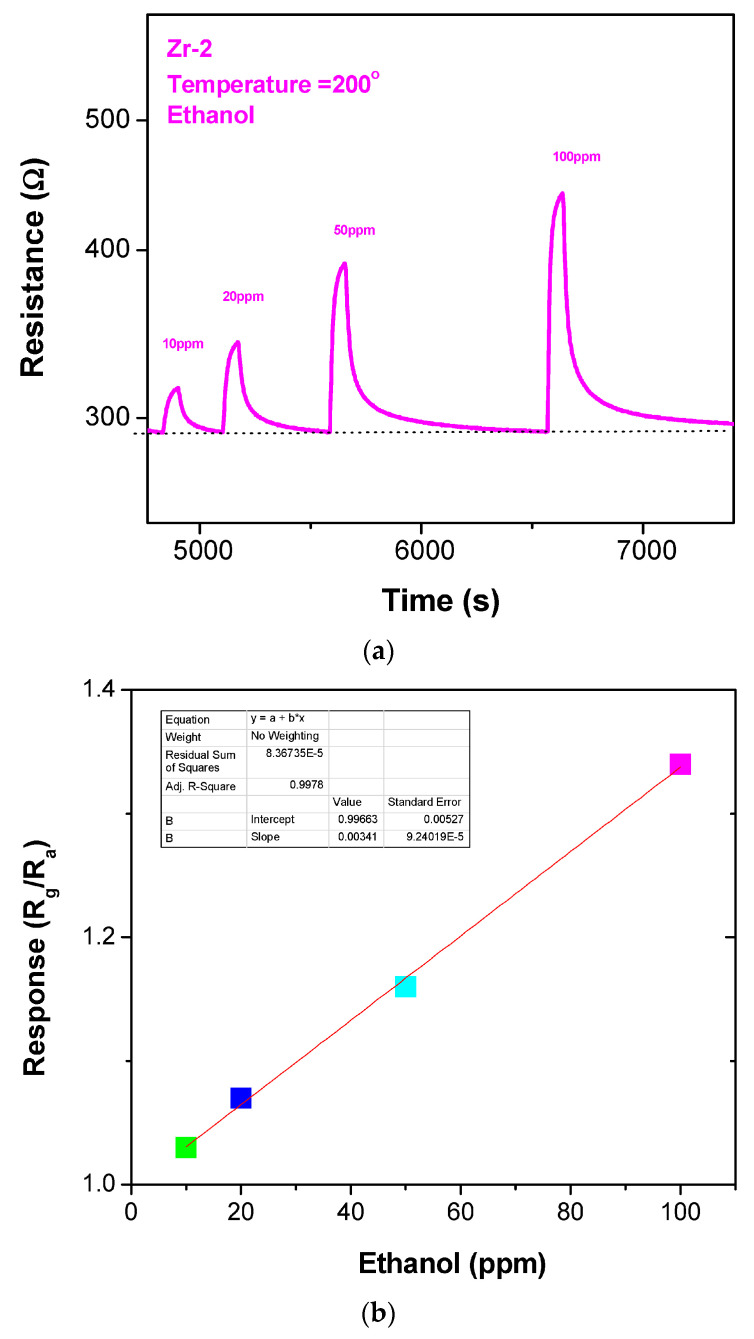
(**a**) Gas-sensing response and (**b**) calibration curve of the ZrO_2_/Co_3_O_4_ nanocomposite sensor to 200 °C under different ethanol concentrations.

After finding the optimal temperature with the right balance between a higher response and faster dynamics, it was set at 200 °C. Figure 6a shows the transient response of the ZrO_2_/Co_3_O_4_ nanocomposite sensor to different amounts of ethanol at the optimum temperature of 200 °C. The response of the sensor increased with an increasing ethanol concentration. This could be because more ethanol molecules participate in the surface reaction, which leads to an amplification of the sensor signal.

In Figure 6b, the calibration curve of response (R_g_/R_a_) vs. ethanol concentration (ppm) is shown. It shows that the increase in the injection of ethanol at a different ppm causes the response to increase continuously. The same tests have been performed with the ZrO_2_ and Co_3_O_4_ sensors. As previously pointed out, the ZrO_2_ showed a very high resistance, so we were not able to obtain any reliable result in the planned experimental conditions. For the Co_3_O_4_ sensor, at the temperature of 200 °C, we registered a signal response to 100 ppm of ethanol, but it was almost negligible (<1.05). The behavior observed in the single ZrO_2_ and Co_3_O_4_ sensors can be ascribed to the high resistance baseline in dry air, which introduces elevated noise and, consequently, low signal stability, which is not suitable for reliable use.

The obtained results demonstrate that the sensor based upon the ZrO_2_/Co_3_O_4_ nanocomposite was more sensitive toward ethanol at 200 °C at low concentrations than the sensors based on the simple metal oxides. The limit of detection (LOD) was 4.85 ppm, as calculated from the calibration curve shown in Figure 6b.

**Figure 7:**
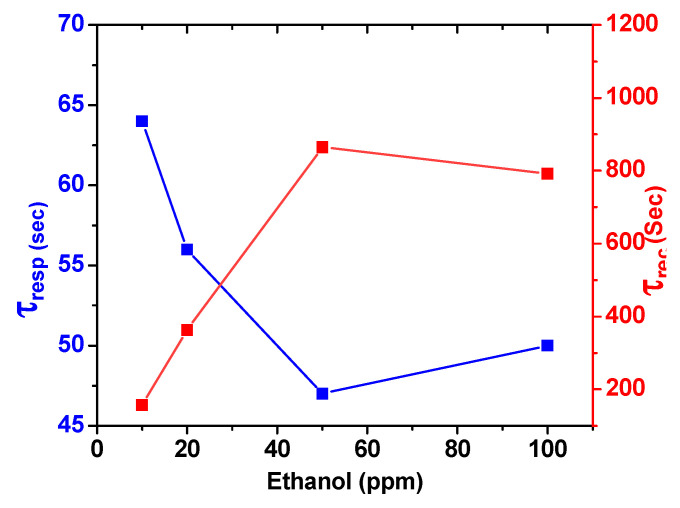
Response (blue) and recovery (red) of the ZrO_2_-Co_3_O_4_ nanocomposite sensor at 200 °C temperature under different ethanol concentrations.

The data in Figure 7 report the response and recovery time of the ZrO_2_/Co_3_O_4_ nanocomposite sensor for different ethanol concentrations at 200 °C. It can be noticed that the sensor displayed a fast response and recovery. The estimated response time at 20 ppm of ethanol was about 56 s and the recovery time at the same concentration of ethanol was 363 s.

**Figure 8:**
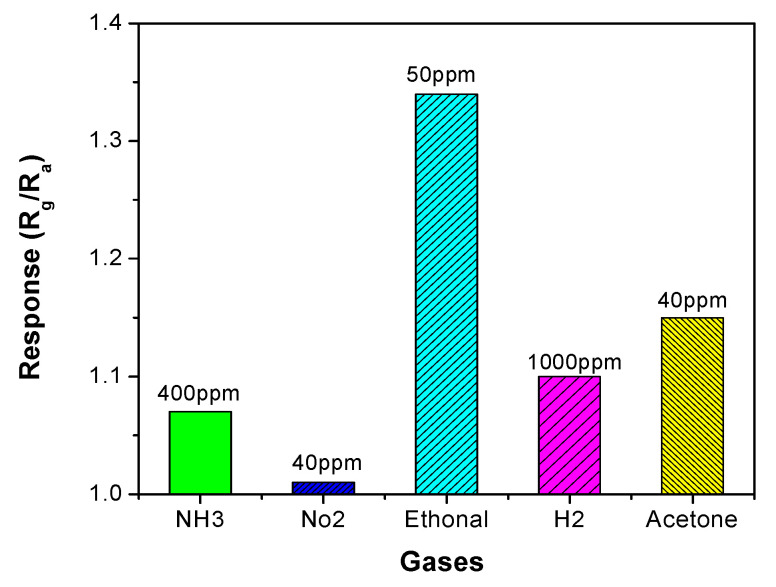
Response of the ZrO_2_-Co_3_O_4_ nanocomposite sensor operating at 200 °C under different gases.

To further validate the performance of the ZrO_2_/Co_3_O_4_ nanocomposite sensor, the selectivity towards the target gas at an operating temperature of 200 °C was investigated. Figure 8 shows the response of the sensor to the gases tested. The response of the sensor to ethanol was higher than that of the other gases (NO_2_, H_2_, acetone, and NH_3_). Table 1 lists the results of the previous literature sensors for ethanol and our composite sensor. Given these results, it can be concluded that the ZrO_2_/Co_3_O_4_ nanocomposite sensor has promising properties. Moreover, it compares very well with the previously developed Co_3_O_4_ sensor and the NiO/Co_3_O_4_ nanocomposite sensor oxides derived from the metal–organic-framework-based sensors for ethanol monitoring.

### 3.3. Gas-Sensing Mechanism

The sensing data indicated that, differently from the sensors based on single Co_3_O_4_ and ZrO_2_ metal oxides, in the conditions adopted, the hybrid nanocomposite sensor demonstrated better performances in response to ethanol in the air. To explain this, we should consider first the general aspect of the gas-sensing mechanism in metal oxides and then consider the aspects related to the change in the chemical, physical, and electronic properties of the hybrid ZrO_2_/Co_3_O_4_ nanocomposite.

The interaction of oxygen with the surface of the sensing layer is the predominant factor that influences the sensing mechanism, and this process is temperature-dependent. When the sensor is exposed to air, oxygen molecule species (from O_2_^−^ to O^2−^, increasing the temperature) adsorb on the surface. At the temperature of 200 °C, the most predominantly adsorbed oxygen species is O^_^. This influences the majority carriers (holes) present in the depletion layer at the grain surface of the p-type semiconductor oxides and, consequently, the resistance baseline. Our results have demonstrated that the composite sensor benefits from this, allowing it to operate at a mild temperature (200 °C). At this temperature, when the composite-based sensor is exposed to ethanol, it adsorbs on the surface of the sensing layer, reacting with the adsorbed oxygen species O^_^, causing a decrease in hole concentration and, consequently, an increase in the resistance, as observed (see Figure 5 and Figure 6).

The better response of the hybrid ZrO_2_/Co_3_O_4_ nanostructure to ethanol compared to the simple oxides can be ascribed to more surface defects that promote gas molecule adsorption and reaction between the adsorbed oxygen species and the target gas. These more active sites come from the electronic interaction between the two p-type metal oxide semiconductors, which create lattice oxygen enrichment and, consequently, boost the reactivity. This hypothesis is supported by our previous research on a p–p type CuO-Co_3_O_4_ heterostructure in the detection of NH_3_ in the air [43] and by other authors on similar composite systems [44].

## 4. Conclusions and Future Perspective

A simple sol-gel method was adopted to synthesize a ZrO_2_/Co_3_O_4_ composite material. The composition, morphology, and structural properties of this material were investigated using different diagnostic techniques, such as XRD, SEM-EDX, Raman, and FT-IR spectroscopies. The sensor prepared using the ZrO_2_/Co_3_O_4_ composite exhibited a good response at the selected working temperature. Low recovery and response times (56 and 363 s, respectively) were obtained at 20 ppm of ethanol concentration, with the ZrO_2_/Co_3_O_4_ sensor, at 200 °C. Furthermore, the ZrO_2_/Co_3_O_4_-based sensor, in the presence of different gases, was highly selective for ethanol. The sensing performance was mainly ascribed to the following factors: (1) the adsorption/desorption of active adsorbed oxygen molecules (e.g., O^−^ and O^2−^) and abundant oxygen vacancies, which increased the number of active sites; (2) the catalytic activity of Co^3+^, which greatly increased the reaction route and decreased the activation energy; and (3) the effective diffusion of gas molecules. Therefore, ZrO_2_/Co_3_O_4_ provides a new material perspective, as well as the engineering of an innovative and low-cost sensing device.

However, future efforts should be focused on enhancing the overall sensor response, optimizing the synthesis procedure, avoiding the presence of synthesis residues, and trying to reduce the sensor working temperature. Indeed, our aim is to reduce power consumption and increase the long-term stability and lifetime of the device.

## Figures and Tables

**Table 1:** Comparison of the previous literature sensors for ethanol with our composite-based sensor.

Sensor	Temp. (°C)	Ethanol ppm	Response Time (s)	Recovery Time (s)	Response (Rg/Ra)	Ref.
CuO-ZnO	350	100	3.2	2.8	7.5	[38]
TiO_2_/SnO_3_	200	10,000	60	50	12.27	[39]
Bi_2_O_3_-In_2_O_3_	200	200	24	180	171	[40]
CdO/ZnO	275	24	NA	NA	29.11	[41]
MoS_2_/TiO_2_	350	100	52	155	NA	[42]
Co_3_O_4_/Fe_2_O_3_	250	100	95	305	1.7	[4]
ZrO_2_/Co_3_O_4_	200	20	56	363	1.07	This work

## Data Availability

Data are contained within the article.
